# “Investor attention fluctuation and stock market volatility: Evidence from China”

**DOI:** 10.1371/journal.pone.0293825

**Published:** 2023-11-27

**Authors:** Taiji Yang, Siqi Zhuo, Yongsheng Yang

**Affiliations:** Pan-Asia Business School, Yunnan Normal University, Kunming, China; The University of Hong Kong, HONG KONG

## Abstract

This paper examines the linkage between Chinese stock market volatility and investor attention fluctuation. In Heterogeneous autoregressive (HAR) model, first, we analyzed the linkage between both decomposed and undecomposed stock market realized volatility and investor attention fluctuations across full-sample and two-year moving window sub-samples. Second, we compare the predictive power of four models in short-, medium-, and long-term volatility forecasting. Empirical results show large positive attention fluctuation amplified Chinese stock market volatility after the outbreak of COVID-19, and negative small attention fluctuation significantly stabilized stock market volatility before COVID-19, and the impact dwindled in after COVID-19. The model incorporating decomposed realized volatility and decomposed attention fluctuation performs better in volatility Forecasting. This research underscores a shift in the dynamics between stock market volatility and investor attention fluctuations, and investor attention fluctuation improves the volatility forecasting accuracy of the Chinese stock market.

## 1. Introduction

Each oscillation within the stock market can be attributed to the psychology of investors. The stock market, serving as a platform for capital distribution, reflects a nation’s political, economic, and cultural landscape. Although Chinese stock market ranks as the second largest market globally, it is predominantly comprised of individual investors. The attention fluctuations within this group of investors can significantly influence market dynamics. Therefore, to invigorate the capital market and foster sound development of the Chinese stock market, it is crucial to augment the exploration of the relationship between investor attention fluctuation and market volatility in Chinese stock market.

Simon [1] introduced the concept of ’limited attention’ to describe the limited energy and time of investors, making it impossible for them to properly process information about the stock market in a timely and sufficient manner, resulting in stock prices not fully reflecting all available information. Traditional methods of measuring investor attention are limited. Post-2000, researchers have utilized proxies such as stock trading volume [2], turnover rate [3], and advertising fees to gauge investor attention. Both trading volume and turnover rate are viewed from the lens of asset transactions, and are also employed as indicators of investor sentiment. However, advertising expenditures, representing merely one facet that attract investor attention, does not provide a comprehensive measure of overall investor attention. Thus, these indicators are not the most effective indicators of investor attention. With the development of the internet, researchers have increasingly employed internet search volume and social media discussions extensively to measure investor attention, finding that integration of internet data enhances the predictive accuracy of models. For instance, Da, Engelberg, and Gao [4] used Google Trends as a proxy for investor attention, arguing that higher search volume denotes more investor attention. Stock forums, as highlighted by studies of Antweiler and Frank [5] and Das and Chen [6], have emerged as crucial sources of information influencing investors’ decisions.

Andrei and Hasler [7] pioneered in emphasizing the relationship between increased investor attention and heightened stock return volatility. El Ouadghiri et al. [8] further evidenced a strong positive correlation between institutional investor attention and stock market liquidity and volatility. Wang, Xu, and Sharma [9] enriched the literatures by segmenting investor attention and illustrated that expected attention serves as informational to the stock market, while Audrino, Sigrist, and Ballinari [10] revealed the significant accuracy improving impact of investor attention on volatility forecasts. Herwartz and Xu [11] further extended the discourse, highlighting the concurrent impacts on volatilities and trading volumes. Internet search volume can serve as a reliable indicator of investor attention and it is possible to construct investor attention index using internet search volume data.

COVID-19 pandemic has elicited a range of economic responses. Bao and Huang [12] highlighted the role of FinTech in providing liquidity during COVID-19. Lockdowns had a negative impact on household incomes and a significant shift in public sentiment exist as the lockdown progressed [13–15]. Zhou et al. [16] offered insight into how small businesses navigated unexpected shocks during the pandemic in China, underlining the managerial biases during the pandemic may lead to unintended aggregate inefficiency. Regarding stock returns during COVID-19, insights from Yu and Huang [17, 18]; Yu, Huang, and Chen [19] unraveled relationships between cross-sectional uncertainty, option-implied idiosyncratic skewness, and cyclical movements in valuation ratios respectively, with Yu et al. [20] further explored the role of adjusted earnings yield in forecasting dividend growth.

The complexity of stock market dynamics, especially in the face of external shocks like the COVID-19 pandemic, underscores the need to delve deeper into the factors influencing stock market volatility. We primarily investigate the impact of fluctuations in investor attention on the realized volatility (RV) of the Chinese stock market. To address this, we develop an attention index derived from Baidu search volume data and conduct a comprehensive analysis of the range of investor attention fluctuations. We categorize investor attention fluctuations into four levels—small, medium, large, and major—based on varying percentage changes in the attention index.

Regarding contributions of this paper, by estimating the relationship between investor attention fluctuation and stock market realized volatility with HAR model, we can find that positive attention fluctuations exceeding 7% become pivotal in affecting Chinese stock market volatility post the outbreak of COVID-19. Negative attention fluctuations below 3% significantly impact stock market volatility before COVID-19, but this effect dwindles in post the outbreak of COVID-19 pandemic. Moreover, the incorporation of the decomposed investor attention volatility into the model enhances the accuracy of short and medium -term volatility forecasts.

This paper is organized as follows: section 2 discusses the methodology we employed. Section 3 provides statistical characteristics of the stock market realized volatility and internet search volume data. Section 4 discusses the empirical results of the linkage between fluctuations in investor attention and stock market volatility. Section 5 discussed the forecasting power of models. Section 6 of this paper summarizes the conclusions.

## 2. Methodology

### 2.1 Realized volatility

Realized volatility (RV) prompted by Andersen and Bollerslev [21] can be constructed as the sum of squared intraday returns on day *t*, which can be expressed as:

$$RV=\sum_{i=1}^{M}r_{t,i}^{2}$$ Eq 1

Where *r*_*t*,*i*_ is the i^th^ intraday return for day *t*, which can be calculated as ln(*p*_*t*,*i*_)−ln(*p*_*t*,*i*−1_), and *M* denotes the numbers of intraday returns, we employ RV in the empirical analysis which is based on 5 minute intraday returns.

Following Barndorff-Nielsen, Kinnebrock, and Shephard [22], we decompose RV into two semi-variance measures, *RV*^+^ captures the market upturn and *RV*^−^ captures the market downturn, where *RV*^+^ and *RV*^−^ can be computed as:

$$RV_{t}^{+}=\sum_{i=1}^{M}r_{t,i}^{2}(r_{t,i}>0),$$ Eq 2

$$RV_{t}^{-}=\sum_{i=1}^{M}r_{t,i}^{2}(r_{t,i}<0)$$ Eq 3

### 2.2 Investor attention

Following Da, Engelberg, and Gao [4], we construct the attention index of investors based on the Baidu search volume for each constituent stock of Shanghai Stock Exchange 50 Index (SSE50), which can be expressed as:

$$AI=\sum_{i}^{N}w_{t}^{d}SVI_{t}^{d}$$ Eq 4

While $w_{t}^{{}_{d}}=\frac{MV_{t}^{{}_{d}}}{\sum_{i=1}^{N}MV_{t}^{{}_{d}}}$, *MV*_*d*_ denotes the total market value of stock *d*, *N* represent the number of stocks in SSE50 and *N* equals 50, *t* refers to the day *t*, and $SVI_{t}^{d}$ refers to the Baidu search volume of stock *d* on day *t*. For instance, the Baidu search volume of Stock “ZhongGuoPingAn (601318)” can be obtained from Baidu Index with the Chinese words “中国平安”.

### 2.3 Investor attention fluctuation

We construct attention fluctuation as the difference between the attention index on day *t* and the mean value of attention index between day *t*−1 and day *t*−20, which can be expressed as:

$$AF_{t}=\left(AI_{t}-Mean(AI_{t-1},\cdots ,AI_{t-20})\right)/Mean(AI_{t-1},\cdots ,AI_{t-20}),$$ Eq 5

and $Mean(AI_{t-1},\cdots ,AI_{t-20})$ refers to the average value of investor attention over the preceding 20 days. To discern the degrees of investors’ attention fluctuation, and comprehend how each level of fluctuation influences the stock market’s volatility, we categorize investors’ attention fluctuation into four levels which are $AF_{t}^{Small}$, $AF_{t}^{Medium}$, $AF_{t}^{L\arg e}$, and $AF_{t}^{Major}$, and the range of four levels of fluctuation is presented in Table 1.

**Table 1:** Range of 4 level of investor attention fluctuation.

$AF_{t}^{Small}$	$-3\%<AF_{t}^{Small}<3\%$
$AF_{t}^{Medium}$	$-7\%<AF_{t}^{Medium}<-3\%\cup 3\%<AF_{t}^{Medium}<7\%$
$AF_{t}^{L\arg e}$	$-10\%<AF_{t}^{L\arg e}<-7\%\cup 7\%<AF_{t}^{L\arg e}<10\%$
$AF_{t}^{Major}$	$AF_{t}^{Major}<-10\%\cup 10\%<AF_{t}^{Major}$

### 2.4 Model specification

We employ a Heterogeneous Autoregressive (HAR) model to investigate the relationship between investor attention fluctuation and stock market volatility. Based on heterogeneous market hypothesis of Müller et al. [23]. Corsi [24] used a long lagged autoregressive process, called the heterogeneous autoregressive (HAR) model. The original HAR model specifies RV as a linear function of daily, weekly, and monthly average realized volatility components, which can be:

$$RV_{t+1}=\beta_{0}+\beta_{d}RV_{t}^{d}+\beta_{w}RV_{t}^{w}+\beta_{m}RV_{t}^{m}+\varepsilon$$ Eq 6

Where $RV_{t}^{d}$, $RV_{t}^{w}$ and $RV_{t}^{m}$ being daily, weekly, and monthly average realized volatility components respectively.

The HAR model transforms a basic AR model by imposing distinct restrictions on the autoregressive coefficients of the AR model, facilitating easy estimation through OLS. The ease of estimation coupled with superior performance makes the HAR model a favored choice for forecasting realized volatility.

We incorporate investor attention fluctuation into a HAR model specification to proceed the analysis, the proposed HAR-AF model is as follows in Eq 7. And we substituted the independent variables with positive and negative components, which is as follows in Eq 8.


$$\begin{array}{l} RV_{t+1}=\beta_{0}+\sum_{i\in (d,w,m)}\beta_{1}^{i}RV_{t}^{i}{}^{Stock}+\beta_{2}^{i}AF_{t}^{i}{}^{Small}+\beta_{3}^{i}AF_{t}^{i}{}^{Medium}\\ \;+\beta_{4}^{i}AF_{t}^{i}{}^{L\arg e}+\beta_{5}^{i}AF_{t}^{i}{}^{Major}+\varepsilon_{t} \end{array}$$ Eq 7

$$\begin{array}{l} RV_{t+1}=\beta_{0}+\sum_{i\in (d,w,m)}\beta_{1}^{i}RV_{t,Stock}^{+,i}+\sum_{i\in (d,w,m)}\beta_{2}^{i}RV_{t,Stock}^{-,i}+\beta_{3}^{}AF_{t}^{+}{}^{Small}\\ \;+\beta_{4}^{}AF_{t}^{-}{}^{Small}+\beta_{5}^{}AF_{t}^{+}{}^{Medium}+\beta_{6}^{}AF_{t}^{-}{}^{Medium}+\beta_{7}^{}AF_{t}^{+}{}^{L\arg e}+\beta_{8}^{}AF_{t}^{-}{}^{L\arg e}\\ \;+\beta_{9}^{}AF_{t}^{+}{}^{Major}+\beta_{10}^{}AF_{t}^{-}{}^{Major}+\varepsilon_{t} \end{array}$$ Eq 8

Where *i* denotes the measures are the daily and weekly and monthly averages, the weekly and monthly RV can be calculated as $\frac{1}{5}\sum_{t=1}^{5}RV_{t}$, $\frac{1}{22}\sum_{t=1}^{22}RV_{t}$ respectively. *RV*_*t*,*stock*_ denotes stock market realized volatility, and *AF*^*Small*^, *AF*^*Medium*^, *AF*^*L*arg*e*^, *AF*^*Major*^ denote small, medium, large, and major investor attention fluctuations respectively, Superscript “+” and “-” denote the positive (*AF*,*RV*>0) and negative (*AF*,*RV*<0) attention fluctuation and RV respectively.

We estimate Eq 8 with a two-year rolling window to ascertain whether the relationship between stock market volatility and investor attention fluctuations varies across different time periods.

## 3. Data

We focus on the Shanghai Stock Exchange 50 Index (SSE50), and Baidu Index data from 10 May 2017 to 30 December 2022, total 1375 trading days. Our choice of SSE50 as a proxy for the Chinese stock market stems from its composition of the top 50 stocks, distinguished by the highest trading volume and capitalization on the Shanghai Stock Exchange. The constituents of SSE50 typically adjust semi-annually. Representing China’s core assets, the SSE50 has been frequently employed in existing literature examining Chinese stock market volatility. Following Da [4], we adopt Baidu Index as a proxy for investor attention, given that Baidu is the largest search engine in China and serves as a principal information source for a majority of investors in the country. The SSE50 RV data can be obtained from CSMAR (https://cn.gtadata.com/), and Baidu Index data can be obtained from Baidu Index (https://index.baidu.com/).

Table 2 presents the statistical description of the variables.

**Table 2:** Statistic descriptions.

	Observations	Mean	Min	Max	Std.Dev.	Skew	Kurtosis	ADF
RV	1375	0.952	0.069	10.936	0.856	3.830	24.956	0.01
Attention index	1375	8697.507	3464.538	15925.669	2674.585	0.707	-0.316	0.01
Small fluctuation	395	—	-0.030	0.030	—	—	—	
Medium fluctuation	416	—	-0.070	0.069	—	—	—	
Large fluctuation	223	—	-0.100	0.098	—	—	—	
Major fluctuation	341	—	-0.367	0.722	—	—	—	

Note: small fluctuation, medium fluctuation, large fluctuation and major fluctuation represent 4 different level of changes in investor attention respectively. We conduct ADF-test to RV and Attention index, both of the p-value of these two variables are less than 0.01, which indicates they are stationary.

**Fig 1:**
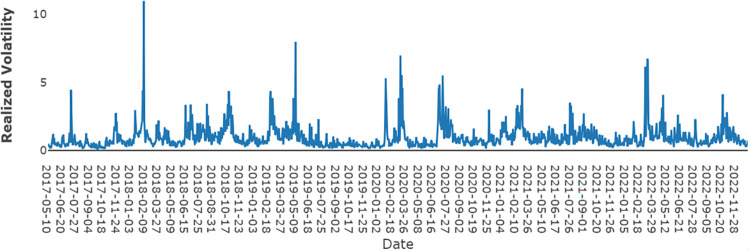


**Fig 2:**
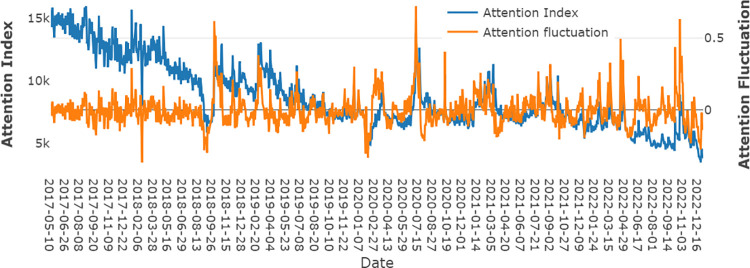


Fig 1 presents the RV of SSE 50 and Fig 2 presents the plot of the investor attention index alongside attention fluctuation. Within Fig 2, the blue line signifies the attention index, and the yellow line represents the attention fluctuation. We can find that RV of SSE50 increase when there is a sharp increase or decrease in investor attention fluctuation. Thus, the study of the relationship between Stock market realized volatility and attention fluctuation is of vital importance for both investors and governors in China.

## 4. Empirical results

### 4.1. In-sample analysis

#### 4.1.1. Full-sample result

The full-sample results are shown in Table 3. The second and third columns show the results when investor attention fluctuations are excluded and included respectively.

**Table 3:** Full sample result.

	Without AF	With AF
(Intercept)	0.2668***	0.2673***
*Std*.*err*	-0.04	-0.04
Daily RV	0.2765***	0.2271***
*Std*.*err*	-0.03	-0.03
Weekly RV	0.4536***	0.4263***
*Std*.*err*	-0.05	-0.05
Monthly RV	-0.0101	0.0736
*Std*.*err*	-0.05	-0.06
Small Fluctuation		-1.5344
*Std*.*err*		-2.01
Medium Fluctuation		1.0917
*Std*.*err*		-0.71
Large Fluctuation		1.0991*
*Std*.*err*		-0.57
Major Fluctuation		0.9653***
*Std*.*err*		-0.22
Adj.R-Squared	0.323623	0.331997

Note: “AF” means investor attention fluctuation.

***, ** and * indicate the result is significant at the 1%, 5% and 10% levels, respectively.

We can find that daily and weekly average volatility measures are highly significant in both models underscoring the persistence of market volatility. Column 3 reveals that small fluctuation component exerts a negative impact on market volatility whereas the other three components have positive impact on market volatility (with the impacts of the large and major fluctuation components being significant). This suggests that small fluctuations stabilize the stock market while medium, large, and major fluctuations increase market volatility in full sample. This phenomenon may attribute to investor herding behavior. People purchase stocks as market fervor markedly escalates, consequently leading to increased volatility.

Table 4 presents the full-sample results with RV, RV+, and RV- as explained variable. The results show that large and major positive attention fluctuations (above 7%) can do a significant positive impact on stock market volatility, while negative ones can only do little and insignificant impacts, people make decisions when heat of the index raise pronouncedly, but do nothing while it drops.

**Table 4:** Full sample results with RV, RV+, RV- as explained variable.

Explained Variable	RV	RV+	RV-
(Intercept)	0.2127***	0.1236***	0.0891**
*Std*.*err*	-0.06	-0.04	-0.03
Positive Small Fluctuation	1.9224	0.7274	1.195
*Std*.*err*	-3.97	-2.44	-2.19
Negative Small Fluctuation	-4.2945	-2.4615	-1.833
*Std*.*err*	-3.7	-2.28	-2.04
Positive Medium Fluctuation	1.416	1.0268	0.3892
*Std*.*err*	-1.58	-0.97	-0.87
Negative Medium Fluctuation	0.0327	-0.2365	0.2693
*Std*.*err*	-1.32	-0.81	-0.73
Positive Large Fluctuation	3.1477***	1.3428*	1.8049***
*Std*.*err*	-1.18	-0.72	-0.65
Negative Large Fluctuation	-0.3965	0.0242	-0.4207
*Std*.*err*	-0.9	-0.55	-0.5
Positive Major Fluctuation	1.5805***	1.0701***	0.5103***
*Std*.*err*	-0.33	-0.21	-0.18
Negative Major Fluctuation	-0.136	-0.189	0.053
*Std*.*err*	-0.47	-0.29	-0.26
Adj.R-Squared	0.339773	0.272083	0.277337

Note: For the sake of brevity, only the results of investor attention fluctuation are displayed.

***, ** and * indicate the result is significant at the 1%, 5% and 10% levels, respectively.

#### 4.1.2. Sub-sample result

Table 5 presents the relationship between decomposed investor attention fluctuation and stock market realized volatility. As observed in Table 5, the absolute value of the coefficient corresponding to negative small attention fluctuation exhibits a declining trend, indicating that it has a strong and significant effect on stabilizing the stock market volatility in the first two subsamples, then the effect remains weak and insignificant in the latter subsamples. The stabilizing effect might be attributed to the general market phenomenon where negative small attention fluctuation could lead to few trading behaviors, thus reducing volatility.

**Table 5:** Subsample results.

	2017–2018	2018–2019	2019–2020	2020–2021	2021–2022
(Intercept)	0.1627	0.1808	0.1558	0.2304**	0.3285***
*Std*.*err*	-0.12	-0.12	-0.1	-0.11	-0.11
Positive Small Fluctuation	4.0032	3.259	2.3809	3.8726	-3.7748
*Std*.*err*	-6.9	-7.44	-7.8	-6.85	-6.17
Negative Small Fluctuation	-12.3719*	-11.9727*	-2.1221	-0.0563	4.289
*Std*.*err*	-6.55	-6.73	-6.69	-6.24	-6.1
Positive Medium Fluctuation	2.4488	3.1547	0.3263	0.8682	0.5396
*Std*.*err*	-2.96	-2.92	-2.88	-2.44	-2.44
Negative Medium Fluctuation	-0.6145	-0.4317	-0.6105	-1.2075	1.2816
*Std*.*err*	-2.51	-2.33	-2.19	-2.21	-2.28
Positive Large Fluctuation	-0.7691	-0.9725	6.3181***	7.1133***	2.9961*
*Std*.*err*	-2.33	-2.22	-2.11	-1.78	-1.79
Negative Large Fluctuation	-0.5025	-0.2096	-1.6741	-0.7624	1.1738
*Std*.*err*	-1.77	-1.73	-1.61	-1.42	-1.4
Positive Major Fluctuation	1.3705**	1.2525**	2.1390***	2.0746***	0.9916*
*Std*.*err*	-0.61	-0.6	-0.61	-0.58	-0.56
Negative Major Fluctuation	0.2395	0.0196	-1.6160*	-0.9564	1.0334
*Std*.*err*	-0.91	-0.93	-0.84	-0.76	-0.78
Adj.R-Squared	0.286099	0.280782	0.357389	0.370594	0.327404

Note: 2017–2018 indicate the time span of our subsample, we chose a rolling 2-year window as a subsample to conduct the analysis. COVID-19 pandemic broke out during 2019-2020.For the sake of brevity, only the results of investor attention fluctuation are displayed.

***, ** and * indicate the result is significant at the 1%, 5% and 10% levels, respectively.

Concerning positive large investor attention fluctuation, the absolute value of its coefficient exhibits a growing trend showing that large positive investor attention exerts weak and insignificant effect on stock market volatility, and its effect grows strong and become significant in the latter 3 subsamples. As for major attention fluctuation, the positive component shows a significant positive impact on market volatility in all subsamples, this aligns with the findings that increased investor attention, especially during significant market events, can lead to heightened market reactions and volatility, aligns with the results of Ballinari, Audrino, and Sigrist [25]; Chen, Tang, et al. [26].

Large positive attention fluctuations are pivotal in increasing Chinese stock market volatility post COVID-19 outbreak. Conversely, small negative attention fluctuations significantly stabilize volatility pre-COVID-19, but this effect dwindles in post the pandemic. An inverse relationship exists between market sensitivity to positive large attention fluctuation and that to negative small attention fluctuation.

The COVID-19 pandemic likely exacerbated market sensitivity to investor attention, particularly as it pertains to pandemic-related news and global economic uncertainties, people trade more when the heat of the stock rise. The pandemic possibly triggered a shift in investor behavior, making investors less reactive to market noise and more responsive to substantial market-impacting events. This behavioral shift could be fueled by the pervasive uncertainty and the global economic impact induced by the pandemic.

Motivated by Gong and Lin [27], we substituted our dependent variable with positive stock market realized volatility and negative stock market realized volatility. This adjustment was aimed at examining whether the observed relationship between stock market realized volatility and investor attention fluctuations remains consistent when analyzing the relationship between decomposed market realized volatility and investor attention fluctuations. This analysis is of paramount practical significance for both investors and researchers, as it can provide nuanced insights into market behavior under varying conditions. The outcomes of this examination are delineated in Tables 6 and 7.

**Table 6:** Subsample results using RV+ as an explained variable.

	2017–2018	2018–2019	2019–2020	2020–2021	2021–2022
(Intercept)	0.1454**	0.1198*	0.080	0.1270*	0.1355**
*Std*.*err*	-0.07	-0.07	-0.07	-0.07	-0.07
Positive Small Fluctuation	0.611	-0.676	3.503	5.212	-3.842
*Std*.*err*	-3.84	-4.16	-5.2	-4.73	-3.72
Negative Small Fluctuation	-3.834	-4.852	-3.038	-3.022	1.679
*Std*.*err*	-3.65	-3.76	-4.46	-4.3	-3.68
Positive Medium Fluctuation	1.202	2.213	1.079	0.824	0.209
*Std*.*err*	-1.65	-1.63	-1.92	-1.68	-1.47
Negative Medium Fluctuation	0.202	0.100	-1.150	-1.875	0.561
*Std*.*err*	-1.4	-1.3	-1.46	-1.52	-1.37
Positive Large Fluctuation	-1.052	-0.879	3.9928***	4.5165***	0.651
*Std*.*err*	-1.3	-1.24	-1.41	-1.22	-1.08
Negative Large Fluctuation	0.300	0.360	-1.011	-0.848	1.043
*Std*.*err*	-0.98	-0.97	-1.07	-0.98	-0.84
Positive Major Fluctuation	1.0109***	0.7672**	1.3918***	1.6231***	0.7231**
*Std*.*err*	-0.34	-0.33	-0.41	-0.4	-0.34
Negative Major Fluctuation	0.144	0.091	-1.3404**	-1.1892**	0.726
*Std*.*err*	-0.51	-0.52	-0.56	-0.52	-0.47
Adj.R-Squared	0.255	0.246	0.231	0.249	0.302

Note: 2017–2018 indicate the time span of our subsample, we chose a rolling 2-year window as a subsample to conduct the analysis. COVID-19 pandemic broke out during 2019-2020.For the sake of brevity, only the results of investor attention fluctuation are displayed.

***, ** and * indicate the result is significant at the 1%, 5% and 10% levels, respectively.

**Table 7:** Subsample results using RV- as an explained variable.

	2017–2018	2018–2019	2019–2020	2020–2021	2021–2022
(Intercept)	0.017	0.061	0.076	0.1034*	0.1930***
*Std*.*err*	-0.07	-0.07	-0.05	-0.06	-0.07
Positive Small Fluctuation	3.393	3.935	-1.122	-1.340	0.067
*Std*.*err*	-3.97	-4.17	-3.81	-3.63	-3.72
Negative Small Fluctuation	-8.5375**	-7.1206*	0.916	2.966	2.610
*Std*.*err*	-3.77	-3.77	-3.27	-3.31	-3.68
Positive Medium Fluctuation	1.247	0.942	-0.753	0.045	0.330
*Std*.*err*	-1.7	-1.64	-1.41	-1.3	-1.47
Negative Medium Fluctuation	-0.817	-0.532	0.540	0.668	0.721
*Std*.*err*	-1.45	-1.31	-1.07	-1.17	-1.37
Positive Large Fluctuation	0.283	-0.093	2.3253**	2.5968***	2.3448**
*Std*.*err*	-1.34	-1.25	-1.03	-0.94	-1.08
Negative Large Fluctuation	-0.802	-0.569	-0.663	0.086	0.131
*Std*.*err*	-1.02	-0.97	-0.79	-0.75	-0.84
Positive Major Fluctuation	0.360	0.485	0.7472**	0.452	0.269
*Std*.*err*	-0.35	-0.33	-0.3	-0.31	-0.34
Negative Major Fluctuation	0.096	-0.072	-0.276	0.233	0.308
*Std*.*err*	-0.53	-0.52	-0.41	-0.4	-0.47
Adj.R-Squared	0.215963	0.2224	0.367067	0.325077	0.193009

Note: 2017–2018 indicate the time span of our subsample, we chose a rolling 2-year window as a subsample to conduct the analysis. COVID-19 pandemic broke out during 2019-2020.For the sake of brevity, only the results of investor attention fluctuation are displayed.

***, ** and * indicate the result is significant at the 1%, 5% and 10% levels, respectively.

The results presented in Tables 6 and 7 are broadly consistent with the ones we get in Table 4, investors’ sensitivity to the stock market had changed, and investors tend to pay more attention to real shocks.

Our analysis predominantly revolves around the SSE 50 Index, which encapsulates the performance of 50 major companies listed on the Shanghai Stock Exchange. While this index reflects a significant portion of the market capitalization and trading activity, it does not entirely represent the broader dynamics and the diverse range of stocks within the entire Chinese stock market.

### 4.2 Out of sample analysis

Forecasting accuracy is also an essential aspect. We employ Eqs 7 and 8 to conduct short, medium, and long term out-of-sample forecasting analysis for market realized volatility, we select July 23, 2021, to December 30, 2022, in total 350 trading days as test data for model comparison.

#### 4.2.1 Evaluation method

We apply 3 common loss functions to do the first comparison, namely the MSE, MAE and QLIKE loss function (defined in Eq 9, Eq 10 and Eq 11),

$$MSE=n^{-1}{\sum_{t=1}^{n}\left({\overset{⏜}{RV_{t}}}^{p}-RV_{t}\right)}^{2}$$ Eq 9

$$MAE=n^{-1}\sum_{t=1}^{n}\left|{\overset{⏜}{RV_{t}}}^{p}-RV_{t}\right|$$ Eq 10

QLIKE=n−1∑t=1n(Ln(RVt⏜p)+RVtRVt⏜p) Eq 11

${\overset{⏜}{RV_{t}}}^{p}$ represents the predict value from models and *RV*_*t*_ represents the actual value of realized volatility. The results are presented in Tables 8–10 respectively.

**Table 8:** One -step ahead forecasting results.

	MSE	QLIKE	MAE
RV only	0.474	1.185	0.433
Decomposed RV	0.457	1.177	0.425
RV and Attention fluctuation	0.430	1.148	0.413
Decomposed RV and Decomposed Attention fluctuation	**0.418**	**1.143**	**0.408**

Note: “RV” means stock market realized volatility is included in the model, “Decomposed RV” means positive and negative component of realized volatility are included in the model. “Attention fluctuation” means investor attention fluctuation is included in the model, “Decomposed Attention fluctuation” means positive and negative component of investor attention fluctuation are included in the model.

**Table 9:** Five -step ahead forecasting results.

	MSE	QLIKE	MAE
RV only	0.570	1.490	0.457
Decomposed RV	0.551	1.402	0.448
RV and Attention fluctuation	0.564	1.448	0.459
Decomposed RV and Decomposed Attention fluctuation	**0.542**	**1.376**	**0.447**

Note: “RV” means stock market realized volatility is included in the model, “Decomposed RV” means positive and negative component of realized volatility are included in the model. “Attention fluctuation” means investor attention fluctuation is included in the model, “Decomposed Attention fluctuation” means positive and negative component of investor attention fluctuation are included in the model.

**Table 10:** Twenty-two steps ahead forecasting results.

	MSE	QLIKE	MAE
RV only	0.615	1.579	0.473
Decomposed RV	0.600	1.472	0.473
RV and Attention fluctuation	0.589	1.514	**0.465**
Decomposed RV and Decomposed Attention fluctuation	**0.572**	**1.451**	0.468

Note: “RV” means stock market realized volatility is included in the model, “Decomposed RV” means positive and negative component of realized volatility are included in the model. “Attention fluctuation” means investor attention fluctuation is included in the model, “Decomposed Attention fluctuation” means positive and negative component of investor attention fluctuation are included in the model.

The model incorporating decomposed stock market realized volatility and decomposed attention fluctuation demonstrates superior performance, achieving the lowest MSE, QLIKE, and MAE in short-term and medium-term volatility forecasting, as well as the lowest MSE and QLIKE in long-term volatility forecasting. The superiority of this model in short-term and medium-term forecasting is supported by all three measures, and its long-term forecasting accuracy surpasses that of the other three models.

### 4.3 Robustness check

To ensure the robustness, we construct attention index by changing internet search volume from single volume of stock name to aggregate internet search volume of both stock name and stock ticker symbols. We present the results in Table 11. The results show a similar pattern to the results of Table 5 which suggests that investors are increasingly tuning out market noise and becoming more responsive to substantial events that impact the market.

**Table 11:** Robustness check.

	2017–2018	2018–2019	2019–2020	2020–2021	2021–2022
(Intercept)	0.1878	0.2303*	0.2153*	0.2465**	0.3192***
Std.err	-0.12	-0.12	-0.11	-0.11	-0.12
Positive Small Fluctuation	1.5151	-0.9294	-4.2716	1.3537	-2.107
Std.err	-7.71	-7.95	-9.16	-8.24	-6.49
Negative Small Fluctuation	-10.9138	-8.5791	3.4707	0.3775	4.8057
Std.err	-6.7	-7.01	-6.82	-6.34	-6.25
Positive Medium Fluctuation	1.3458	1.3387	0.9318	1.84	-0.2804
Std.err	-2.85	-2.88	-2.85	-2.54	-2.49
Negative Medium Fluctuation	-0.2868	0.4565	-0.2629	-1.517	1.2378
Std.err	-2.57	-2.42	-2.29	-2.29	-2.34
Positive Large Fluctuation	0.4136	-0.5122	3.2941	4.3019**	3.1073*
Std.err	-2.53	-2.25	-2.06	-1.84	-1.81
Negative Large Fluctuation	0.3874	0.7773	-0.3224	-0.2341	1.0065
Std.err	-1.71	-1.65	-1.62	-1.48	-1.4
Positive Major Fluctuation	1.3089**	1.1255*	2.0989***	2.2850***	1.2288**
Std.err	-0.66	-0.63	-0.63	-0.6	-0.57
Negative Major Fluctuation	0.4374	0.392	-1.4648	-0.9726	1.216
Std.err	-0.96	-0.98	-0.89	-0.8	-0.81
Adj.R-Squared	0.283406	0.27759	0.353476	0.361326	0.3307

Note: The attention index is constructed by aggregate internet search volume of stock name and stock ticker symbols

We next check the robustness of forecasting performance by conducting the model confidence set (MCS) test proposed by Hansen, Lunde, and Nason [28]. Following the methodologies of González-Rivera, Lee, and Mishra [29]; Hansen and Lunde [30], we employ six different loss functions to conduct the MCS test separately. The definitions of these loss functions are presented in Table 12.

**Table 12:** Loss functions.

$MSE1=n^{-1}{\sum_{t=1}^{n}\left({\overset{⏜}{RV_{t}}}^{p}-RV_{t}\right)}^{2}$	$MSE2=n^{-1}{\sum_{t=1}^{n}\left({\overset{⏜}{RV_{t}{}^{2}}}^{p}-RV_{t}{}^{2}\right)}^{2}$
$MAE1=n^{-1}\sum_{t=1}^{n}\left|{\overset{⏜}{RV_{t}}}^{p}-RV_{t}\right|$	$MAE2=n^{-1}\sum_{t=1}^{n}\left|{\overset{⏜}{RV_{t}{}^{2}}}^{p}-RV_{t}{}^{2}\right|$
QLIKE=n−1∑t=1n(Ln(RVt⏜p)+RVtRVt⏜p)	$R2LOG=n^{-1}\sum_{t=1}^{n}{\left[\log(RV_{t}{}^{2}{\overset{⏜}{RV}}^{p}{{}_{t}}^{-2})\right]}^{2}$

${\overset{⏜}{RV_{t}}}^{p}$ represents the predict value from models and *RV*_*t*_ represents the actual value of realized volatility. In MCS test, the results are bootstrapped 10000 times and a confidence level of 0.05 is used. The results of one-step ahead forecasting MCS test, five-step ahead forecasting MCS test, and twenty-two steps forecasting MCS test are presented in Tables 13–15 respectively.

**Table 13:** MCS test results of one-step ahead prediction.

	Loss Function					
	MSE1	MSE2	MAE1	MAE2	QLIKE	R2LOG
RV only	—	—	—	—	—	—
Decomposed RV	—	3	—	—	3	3
RV and Attention fluctuation	—	2	—	2	2	2
Decomposed RV and Decomposed Attention fluctuation	1	1	1	1	1	1

**Table 14:** MCS test results of five-step ahead prediction.

	Loss Function					
	MSE1	MSE2	MAE1	MAE2	QLIKE	R2LOG
RV only	—	4	3	3	—	—
Decomposed RV	2	3	2	1	—	2
RV and Attention fluctuation	3	2	—	4	—	—
Decomposed RV and Decomposed Attention fluctuation	1	1	1	2	1	1

**Table 15:** MCS test results of twenty-two steps ahead prediction.

	Loss Function					
	MSE1	MSE2	MAE1	MAE2	QLIKE	R2LOG
RV only	4	—	3	3	4	4
Decomposed RV	3	—	4	4	1	3
RV and Attention fluctuation	2	—	1	1	3	2
Decomposed RV and Decomposed Attention fluctuation	1	1	2	2	2	1

Sign “—” indicates model is eliminated. MCS test results confirm the superiority of the model incorporating decomposed RV and decomposed attention fluctuation in the short-term and medium-term forecasting. As for long-term forecasting, the model ranks the first under MSE1, MSE2 and R2LOG which still shows attention fluctuation can demystify the characteristics of stock market volatility.

## 5. Conclusion

This paper pioneers the examination of the relationship between Chinese stock market realized volatility and investor attention fluctuation through the lens of the HAR model. Our nuanced analysis across full samples and sub-samples reveals critical insights into the dynamics of investor attention and stock market volatility.

Positive attention fluctuations exceeding 7% are pivotal in influencing Chinese stock market volatility after the outbreak of COVID-19. Conversely, small negative attention fluctuations significantly impact volatility before COVID-19, but this effect dwindles in post pandemic. Notably, an inverse relationship exists between market sensitivity to positive large attention fluctuation and that to negative small attention fluctuation. Our models, encapsulating decomposed Realized Volatility (RV) and decomposed attention fluctuation, demonstrate superior predictive accuracy in forecasting short-term and medium-term realized volatility, as validated by model confidence set tests.

Comprehending this nuanced relationship between investor attention fluctuations and stock market volatility aids investors utilizing attention fluctuations to forecast stock returns and formulate investment strategies. Policymakers can implement more regulations and guidance to steer investors towards value investing and exploit big data for regulatory purposes to mitigate extreme stock market volatility, preventing transmission of financial risks across global markets [31, 32], promoting the healthy development of the financial markets after COVID-19.

Subsequent research could extend the analysis to a more representative array of indices or individual stocks across different market segments to gain a more holistic understanding of the relationship between investor attention fluctuations and market volatility.

Our exploration enriches the behavioral finance discourse, particularly the investor attention effect, and provides empirical evidence of the multifaceted relationship between stock market volatility and varying levels of investor attention fluctuation over different time periods. Additionally, the superior performance of models incorporating decomposed RV and decomposed attention fluctuation in volatility forecasting underscores the potential for advancing financial modeling and risk assessment practices.
